# Use of Oral Polio Vaccine and the Global Incidence of Mother-to-Child Human Immunodeficiency Virus Transmission

**DOI:** 10.3389/fpubh.2022.878298

**Published:** 2022-06-09

**Authors:** Farrokh Habibzadeh, Mahboobeh Yadollahie, Ashraf Simi

**Affiliations:** ^1^Global Virus Network, Middle East Region, Shiraz, Iran; ^2^Freelance Researcher, Shiraz, Iran

**Keywords:** HIV, acquired immunodeficiency syndrome, vertical transmission of infectious disease, oral poliovirus vaccine, cross protection, innate immunity

## Abstract

**Background:**

Mother-to-child transmission (MTCT) of human immunodeficiency virus (HIV) is an important global health issue. We hypothesized that the live attenuated poliovirus existing in oral polio vaccine (OPV) may protect uninfected neonates born to HIV-positive mothers through the stimulation of innate immune system.

**Objective:**

To test the hypothesis that countries using OPV have a lower MTCT rate (due to postnatal protection provided by the vaccine) compared with those using only inactivated polio vaccine (IPV).

**Methods:**

In an ecological study, the incidence of HIV/AIDS in children aged <1 year (IncHIV1), considered a surrogate index for MTCT rate, was compared between countries using OPV vs. IPV. The aggregated population data were retrieved for 204 countries from the Global Burden of Disease (GBD 2019) Collaborative Network website, “Our World in Data” website, the World Bank website, and the WHO Global Polio Eradication Initiative (GPEI). We used a negative binomial regression model with IncHIV1 as the dependent variable and the prevalence of HIV/AIDS in women aged 15–49 years (PrevHIV), antiretroviral therapy (ART) coverage, human development index (HDI), and the type of vaccine used in each country as independent variables. Multivariate imputation by chained equations was used to treat missing values. Analyses were performed for both the original dataset (with missing values) and the five imputed datasets.

**Results:**

IncHIV1 and PrevHIV were available for all 204 countries; vaccine type, 194 countries; HDI, 182 countries; and ART coverage, 133 countries. One-hundred and twenty-nine countries in the original dataset had complete data for all the above-mentioned variables; the imputed datasets had complete data for all 204 countries. The results obtained from the analysis of the original dataset had no overall difference with the pooled results obtained from the analysis of the five imputed datasets. Countries with higher HDI mainly use IPV; those with lower HDI commonly use OPV. PrevHIV, HDI, and the type of vaccine were independent predictors of IncHIV1. Use of OPV compared to IPV, was independently associated with an average decrease of 17% in IncHIV1 at the median HDI of 0.75. The protection provided by OPV increased in countries with lower HDI.

**Conclusions:**

Use of OPV compared with IPV, was independently associated with lower MTCT rate.

## Introduction

Mother-to-child transmission (MTCT) of human immunodeficiency virus (HIV) is an important global health issue. About 1.7 million children aged under 15 years were infected with HIV in 2018 (1). Most of these children were infected through MTCT. If no preventive measure is instituted, 30–45% of infants born to mothers with HIV become infected. However, the rate has significantly dropped to <1% (particularly in high-income countries) after antiretroviral treatments (ARTs) have become available (2). MTCT may occur *in utero*, when the fetus is continuously exposed to maternal viral particles; during labor and delivery, when the newborn becomes in contact with infected maternal birth canal secretions and blood; and after birth, when the infant is exposed to the virus mainly through susceptible cells (e.g., lymphocytes) infected with the virus existing in breast milk (3). Because of long-term daily exposure to breast milk over several months after the birth, the cumulative risk for transmission increases so that breastfeeding accounts for around 40% of all MTCTs (4).

Humoral immune response and passive transfer of maternal antibodies to the infant are among known immune responses for prevention of MTCT (5). The maternal virus is constantly changing to escape the neutralizing antibodies produced. Infants, however, are typically infected with a single variant of the virus, commonly resistant to almost all neutralizing antibodies transferred from their mothers (6). Antibody-dependent cellular cytotoxicity mediated by a variety of effector cells, including natural killer (NK) cells, is also important in the eradication of the virus (5). Cell-mediated immunity has been implicated in prevention of MTCT too (7). But, the first line of defense is provided by the innate immune system (8, 9).

Oral polio vaccine (OPV) has been shown to not only specifically immunize people against polio, but also provide non-specific transient protection against influenza and the severe acute respiratory syndrome coronavirus 2 (SARS-CoV-2) (10–12). In a recent ecological study, we have shown that use of OPV vs. inactivated polio vaccine (IPV) for routine immunization, is associated with about 30% reduction in the incidence of the coronavirus disease 2019 (COVID-19) (13). The protective effect provided by OPV is probably through the stimulation of the innate immune system, as shown earlier for other live attenuated vaccines (LAVs) such as Bacillus Calmette-Guérin (BCG) and measles, mumps, and rubella (MMR) (14, 15).

According to the World Health Organization (WHO) expanded program on immunization (EPI), polio vaccine should be given at birth and at 6, 10, and 14 weeks of age (16). If OPV can provide a non-specific protection against influenza and SARS-CoV-2, then it may provide non-specific protection against other infectious agents such as HIV. We hypothesized that although the innate immune response supposedly produced by OPV has no effect on MTCT rate during the pregnancy and birth, it might prevent the transmission during postnatal period by causing immunity in the neonates who received OPV compared to those receiving IPV. Although this would not abolish the MTCT rate, it would decrease it. Herein, we aimed at indirectly investigate whether receiving OPV can decrease MTCT rate, presumably by preventing postnatal MTCT.

This study was thus conducted to compare the incidence of HIV/AIDS in children aged <1 year (IncHIV1, considered a surrogate index for the MTCT rate) between countries using OPV and IPV. There were, however, many other covariates that might affect the incidence of the disease, including the prevalence of HIV/AIDS in mothers of childbearing age, the quality of the health care system and the surveillance infrastructures, and the availability of ART that would certainly influence the occurrence, reporting, and prevention of MTCT. Therefore, an important part of this study was to provide a model adjusted for the covariates that might influence the conclusions.

## Materials and Methods

### Source of Data

In this ecological study the IncHIV1 and the prevalence of HIV/AIDS in women aged 15–49 years (PrevHIV) were retrieved for each country from the Global Burden of Disease (GBD 2019) Collaborative Network website on 17 December 2021 (17). DisMod-MR 2.1, a Bayesian meta-regression tool, was used in GBD 2019 for estimation and ensuring consistency between the incidence rate and the prevalence reported (18). The country population and population density, the median age and the life expectancy at birth, the gross domestic product (GDP) per capita, and the human development index (HDI) for each country in 2020 were retrieved from “Our World in Data” website on 19 Aril 2021 (19). The ART coverage (percentage of people living with HIV) in 2020 estimated by UNAIDS for each country was retrieved from the World Bank website on 2 January 2022 (20). The type of polio vaccine used in each country in 2020 was provided by the WHO Global Polio Eradication Initiative (GPEI).

### Statistical Analysis

R software version 4.1.0 (2021-05-18) was used for data analysis. Multivariate imputation by chained equations (using the function *mice* of mice package) was used to treat missing values (21). Function *quickpred* of the same package was used to determine the predictor matrix (22). The dependent variable, IncHIV1, was not used for prediction of other variables. PrevHIV was also not used for prediction of other variables, as GBD 19 could estimate it from IncHIV1. Five imputed datasets were generated.

Normal probability plot (using *geom_qq* and *stat_qq* of ggplot2 package) was used to determine if a continuous variable follows normal distribution. Continuous variables were expressed as median (interquartile range [IQR]). Wilcoxon rank sum test (using *wilcox.test* function) was used to compare the distribution of two continuous variables not normally distributed. Pearson's *r* or Spearman's ρ (using *rcorr* function of Hmisc package) was used to determine the extent of correlation between the continuous variables with and without normal distribution, respectively.

Because the IncHIV1 (the dependent variable in our analysis) had overdispersion, a negative binomial regression analysis was used (using *glm.nb* function of MASS package). The median age, life expectancy at birth, and GDP per capita had a high correlation with HDI; thus, only PrevHIV, ART coverage, HDI, and the type of polio vaccine were used as independent variables in our model. Interaction between HDI and the type of polio vaccine was also taken into account. To account for the missing values, we used the imputed datasets in the regression analysis. Outliers were included in all data analyses. The results obtained from analysis of each of the five imputed datasets were then pooled (using function *pool* of mice package). A *p*-value <0.05 was considered statistically significant.

## Results

**Figure 1:**
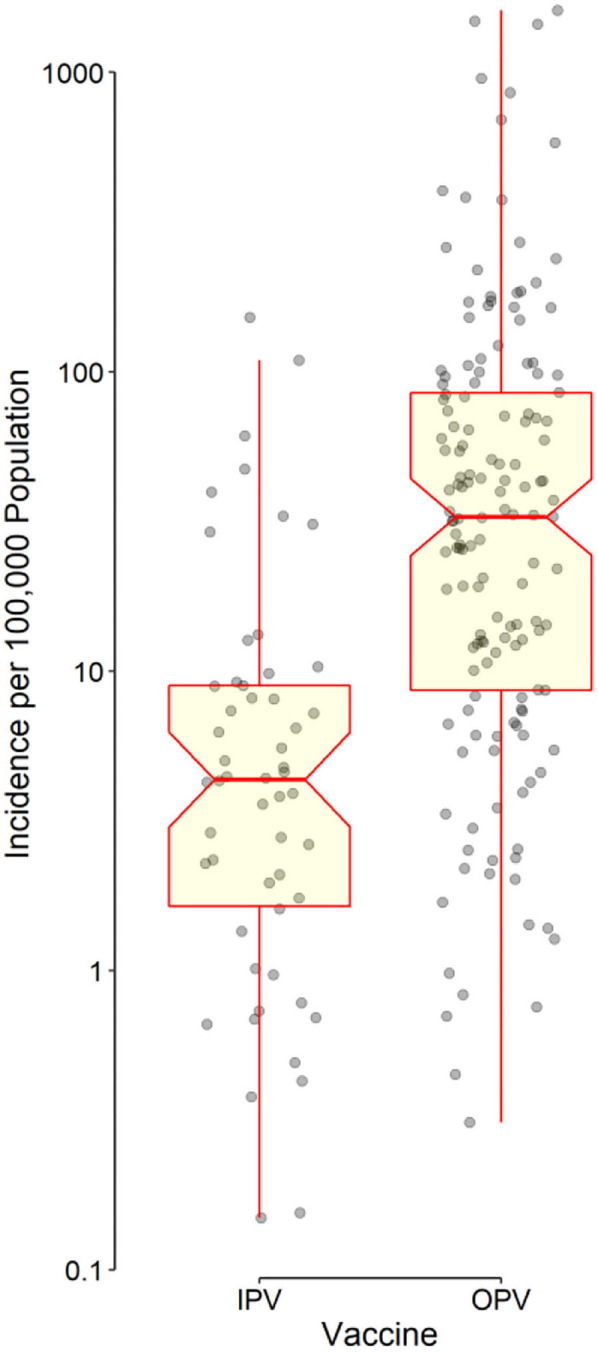
Distribution of data points as well as the box and whisker plot of the incidence of HIV/AIDS in children aged <1 year stratified by the type of polio vaccine used in each country. The horizontal line in the middle of each box indicates the median. The notch represents the 95% confidence interval of the median. The bottom and top borders of the box show the 25th and 75th percentiles, respectively. The lower whisker indicates the smallest data point within 1.5 times the interquartile range (IQR) less than the 25th percentile; the upper whisker indicates the largest point within 1.5 × IQR greater than the 75th percentile. Points greater than the upper whisker and smaller than the lower whisker were considered outliers. All outliers were included in data analyses. Note that the vertical axis has a logarithmic scale.

**Figure 2:**
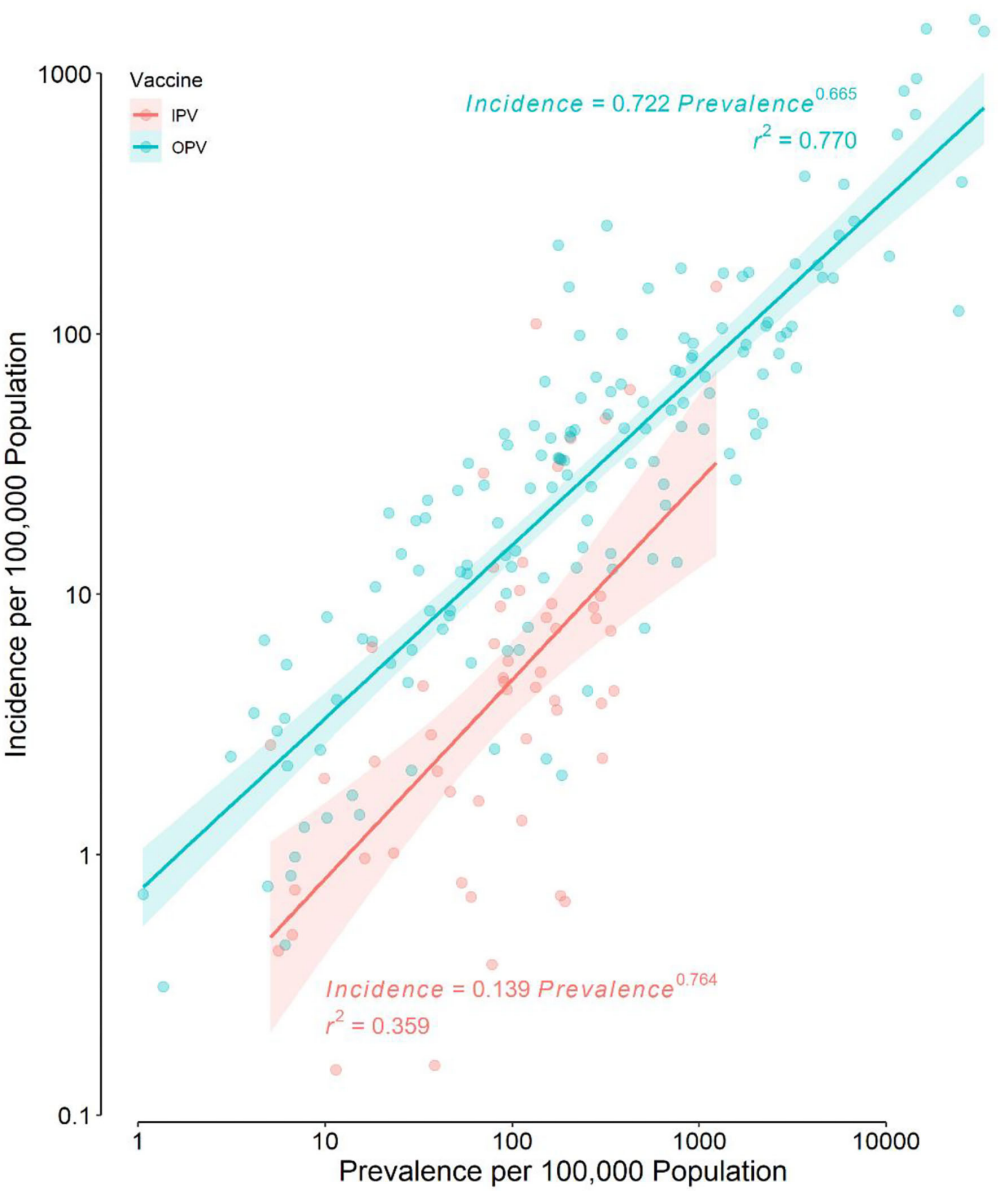
Scatter plot of the incidence of HIV/AIDS in children aged <1 year against the prevalence of HIV/AIDS in women aged 15–49 years in the original dataset. Note that the scale of both axes is logarithmic. The regression lines and their 95% confidence bands are also presented for each type of polio vaccine used.

Distributions of IncHIV1, PrevHIV, and population density became close to normal after log-transformation (Supplementary eFigure 1); distributions of other studied variables were far from normal. Data on the type of polio vaccine used were available for 194 countries—52 used IPV only and 142 used OPV in combination with IPV. ART coverage was available for 133 countries, translating to a missing value percentage of almost 35%. There was no clear pattern of missingness in the dataset (Supplementary eFigure 2), therefore, the assumption of “missing at random” made for multivariate imputation of data was plausible. The distributions of all studied variables, but the population density, were significantly different in countries using OPV compared with IPV only (Table 1). The median IncHIV1 was significantly higher in countries using OPV compared with those using IPV only (Figure 1). There was a positive correlation (*r* = 0.81, *p* < 0.001) between IncHIV1 and PrevHIV after log-transformation of the variables (Figure 2).

**Table 1:** Median (IQR) of studied continuous variables in the original dataset stratified by the type of polio vaccine used.

**Variable**	**Type of polio vaccine used**		***p-*value**
	**IPV (*n* = 52)**	**OPV (*n* = 142)**	
IncHIV1* (per 100,000 population)	4.3 (1.5 to 8.9)	32.9 (8.4 to 85.1)	<0.001
PrevHIV^†^ (per 100,000 population)	110.8 (44.7 to 179.9)	224.5 (47.6 to 1118.9)	0.001
Human development index	0.89 (0.85 to 0.93)	0.70 (0.55 to 0.78)	<0.001
Median age, yrs	41.9 (38.2 to 43.6)	26.7 (25.8 to 31.9)	<0.001
Life expectancy, yrs	81.3 (77.6 to 82.5)	72.0 (65.1 to 75.4)	<0.001
GDP^‡^ per capita, ×1000 $US	35.2 (25.9 to 46.0)	7.5 (2.9 to 14.9)	<0.001
ART^§^ coverage (%)	79.5 (67.0 to 85.0)	55.0 (44.0 to 69.0)	<0.001
Population density (people/km^2^)	105 (38 to 195)	77 (32 to 208)	0.297

^*^Incidence of HIV/AIDS in children aged <1 year; Prevalence of HIV/AIDS in women aged 15–49 years; ^‡^Gross domestic product; ^§^Antiretroviral treatment.

**Figure 3:**
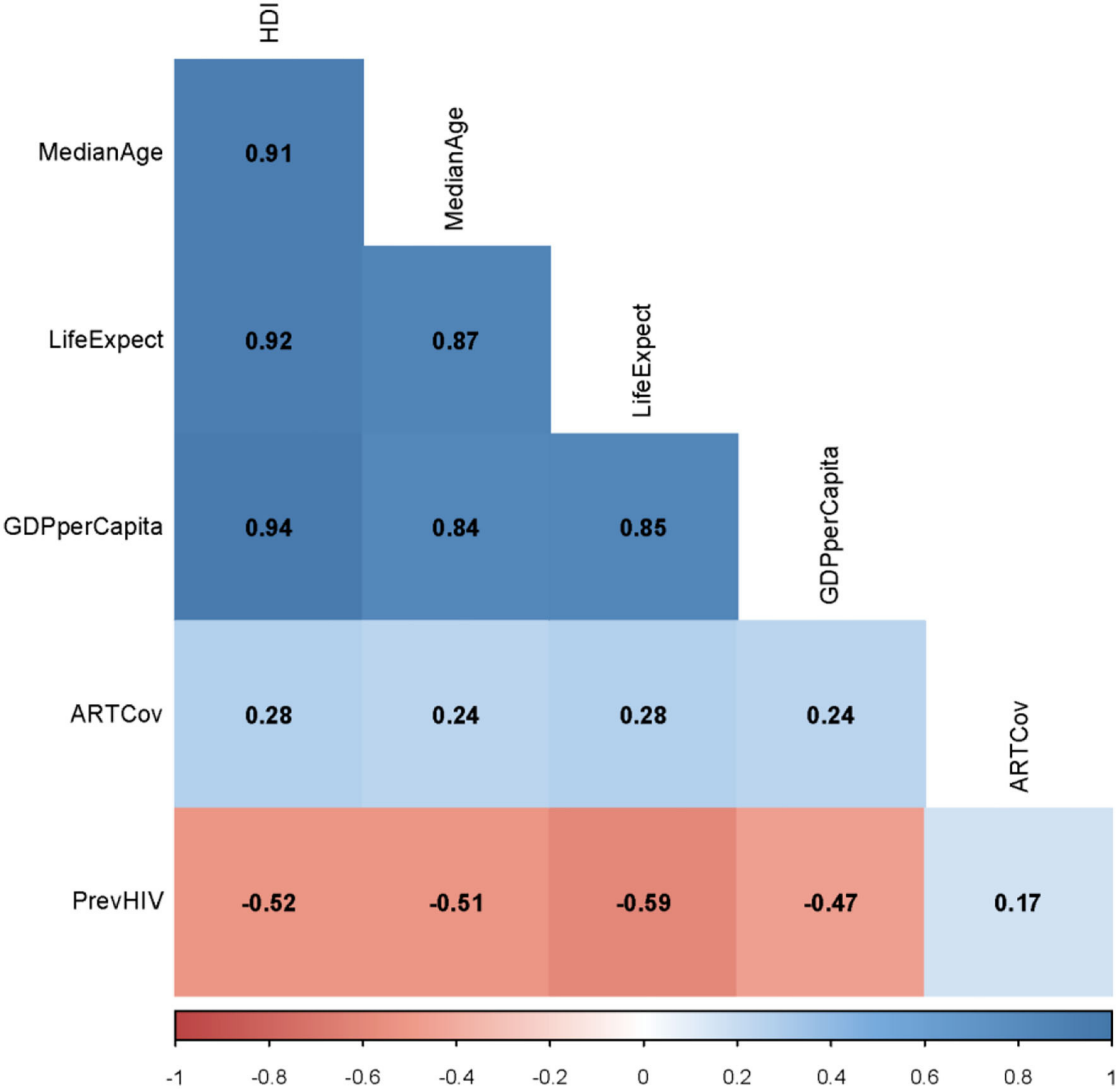
Spearman ρ between each of two studied continuous variables from the original dataset. HDI, human development index; MedianAge, median age; LifeExpect, life expectancy; GDPperCapita, gross domestic product per capita; PrevHIV, prevalence of HIV/AIDS in women aged 15–49 years; and ARTCov, antiretroviral therapy coverage (% of people living with HIV).

There was a significant (*p* = 0.001) correlation between ART coverage and HDI (ρ = 0.28) (Figure 3). The level of correlation between each of the median age, life expectancy at birth, and the GDP per capita and HDI, however, was much higher (ρ > 0.91, *p* < 0.001) (Figure 3). To avoid multicollinearity, we have only used PrevHIV, ART coverage, HDI, and the type of vaccine as independent variables in the regression model. IncHIV1, the dependent variable in our model, had a mean of 81 cases per 100,000 population; the variance was 46,102. For the overdispersion exist in the dependent variable, we used a negative binomial regression. ART coverage did not have any significant correlation with PrevHIV (ρ = 0.17, *p* = 0.052) (Figure 3).

The negative binomial regression model applied to the imputed datasets could explain 85% of the variance observed in IncHIV1 (Table 2). The results obtained from the model applied to the original data (with missing values) are also presented in Table 2. Based on the pooled results, an increase of 1% in PrevHIV was associated with an increase of 19% in IncHIV1 (see Supplementary Materials). The model revealed an interaction between HDI and the type of vaccine used. Each 0.1 unit increase in HDI was associated with a decrease in IncHIV1 by 61% in countries using IPV only and by 22% in countries using OPV (Table 2). Use of OPV compared to IPV was independently associated with an average 17% decrease in the IncHIV1 estimated at the median HDI of 0.75; the protection provided was higher for countries with lower HDI. For example, the value corresponding to an HDI of 0.55, the 25th percentile of HDI in countries using OPV (Table 1), was 70%. ART coverage did not significantly associate with IncHIV1 in our model (Table 2); indeed, it significantly associated with IncHIV1 in none of the models examined (see Supplementary Materials).

**Table 2:** Pooled results of negative binomial regression obtained from the analysis of 5 imputed datasets and the original dataset.

**Variable**	**Dataset**	**Coefficient (95% CI)**	Adj IRR*(95% CI)	***p*-value**
PrevHIV^†^ (per 100,000 population)	Imputed	1.74 (1.36 to 2.12) ×10^−4^	1.00 (1.00 to 1.00)	<0.001
	Original	2.05 (1.44 to 2.79) ×10^−4^	1.00 (1.00 to 1.00)	<0.001
ART^‡^ coverage (%)	Imputed	0.27 (−0.77 to 1.30) ×10^−2^	1.00 (0.99 to 1.01)	0.616
	Original	0.42 (0.52 to 80.90) ×10^−2^	1.00 (0.99 to 1.01)	0.419
Human development index	Imputed	−9.32 (−13.87 to −4.77)	0.90 (0.01 to 84.46) ×10^−4^	<0.001
	Original	−13.84 (−19.25 to −9.16)	0.98 (0.00 to 105.67) ×10^−6^	<0.001
OPV vaccine^§^	Imputed	−5.01 (−9.05 to −0.97)	6.66 (0.12 to 377.93) ×10^−3^	0.018
	Original	−8.77 (−13.67 to −4.55)	1.56 (0.01 to 105.59) ×10^−4^	<0.001
Interaction^¶^	Imputed	6.90 (2.15 to 11.65)	0.99 (0.01 to 114.12) ×10^3^	0.006
	Original	11.67 (6.73 to 17.29)	1.17 (0.01 to 323.97) ×10^5^	<0.001
Intercept	Imputed	10.13 (6.25 to 14.01)	2.50 (0.05 to 121.12) ×10^4^	<0.001
	Original	13.85 (9.78 to 18.63)	1.03 (0.02 to 123.69) ×10^6^	<0.001

^*^Adjusted incidence rate ratio; Prevalence of HIV/AIDS in women aged 15–49 years; ^‡^Antiretroviral treatment; ^§^Using OPV vaccine compared to IPV (reference) (OPV = 1, IPV = 0); ^¶^HDI × Type of vaccine (OPV = 1, IPV = 0); Nagelkerke's R^2^ = 0.85.

## Discussion

The results obtained from applying the regression model to the original data (with missing values) and the imputed datasets were almost similar in terms of the independent predictors identified (Table 2). After adjusting for studied covariates, use of OPV by a country was found to be independently associated with a lower MTCT rate (lower IncHIV1). The median IncHIV1 was significantly lower in countries using IPV only compared with countries using OPV (Table 1; Figure 1). However, this was the result of a univariate analysis and could be affected by other variables. Countries using only IPV typically had a higher HDI and ART coverage compared to those using OPV (Table 1). The observed relatively lower MTCT rate in countries using IPV only might in fact be attributed to the relatively higher HDI and ART coverage in these countries. High-income countries (presumably having higher HDI) could decrease the MTCT rate better than low- and middle-income countries (likely to have relatively lower HDI, commonly using OPV) because of the widespread use of ART in mothers infected with HIV and their babies, doing elective Cesarean section of HIV-positive mothers, and use of formula feeding of their children (23). Treating HIV-infected mothers with antiretroviral agents decreases MTCT by reducing the maternal viral load; the treatment would act as prophylaxis in their children (24). Cesarean section would decrease the time and level of exposure to the virus in situations where access to quality health care facilities is possible, typically in high-income countries (commonly using IPV). However, WHO does not recommend it for low- and middle-income countries where such facilities are not readily available to all people (5, 23, 25). To abolish the risk of MTCT during postnatal period, formula feeding is recommended for children born to HIV-infected mothers. However, this should only be done in high-income countries (commonly using IPV) with a likely better health care infrastructure, ready access to clean potable water, good sanitation, and better ART coverage; WHO recommends that low- and middle-income countries (commonly using OPV) should still use breast feeding because it would prevent infant mortality frequently caused by other infectious diseases and malnutrition (23, 25). Countries with a better health care infrastructure (mostly with a relatively higher HDI) can presumably have better access to diagnostic tests and identify women infected with HIV earlier and treat them in contrast to low- and middle-income countries where some of the HIV-positive women may not even be aware of their infection. This might also explain the irony of observation that the ART coverage did not have any significant contribution to the outcome variable, IncHIV1; it seems that for the significant correlation between the ART coverage and HDI, the effect of ART coverage in our model is exerted by changes in HDI. Countries with higher HDI, expectedly, have provided a better ART coverage.

The incidence of HIV/AIDS in children (IncHIV1) was expectedly positively correlated with the prevalence of HIV/AIDS in mothers of childbearing age (PrevHIV) regardless of the type of polio vaccine used (Figure 2). This positive correlation found in univariate analysis still persisted even after the effects of other variables were controlled in our model (Table 2).

The population density was not significantly different between countries using OPV and IPV only (Table 1). The variable was thus not included in the model. HDI had a strong correlation with the life expectancy at birth, the median age, and GDP per capita in a country (Figure 3). This strong correlation was not surprising; HDI is a measure reflecting the average wellbeing of people in a country and is computed based on the life expectancy at birth (and thus, the median age of the population), gross national income (and thus, the GDP) per capita, and other factors. None of the life expectancy, the median age, and GDP per capita was therefore used in the model.

The type of polio vaccine used in a given country strongly depends on the HDI—countries with higher HDI (commonly, high-income countries) prefer and can afford to use IPV, which is significantly more expensive than OPV. An increase of 0.1 unit in HDI was independently associated with a higher reduction in MTCT rate in countries using IPV only compared with those using OPV (61 vs. 22%, respectively). This observation would be due to the presence of more efficient strategies to combat MTCT in high-income countries including better ART coverage. The strong correlation between the type of the vaccine and HDI might also explain the significant interaction existing between HDI and the type of vaccine used observed in the regression model (Table 2). Moreover, it has been shown that being aware of HIV does not necessarily have a protective effect when HIV-positive women live in poverty compounded with lack of agency typically in low- and middle-income countries usually with a low HDI (commonly using OPV) (26).

Use of OPV compared with IPV only, was associated with a lower MTCT rate (Table 2). The protection provided increased in countries with lower HDI; it increased from 17% for countries with an HDI of 0.75 (the median HDI) to 70% for countries with an HDI of 0.55 (the 25th percentile of HDI for countries using OPV). This might be attributed to the fact that countries with higher HDI (likely to have a better health care infrastructure) have already implemented measures to decrease MTCT, thus, the protective effect of OPV might be less pronounced for them compared to those with lower HDI (presumably having less-developed health care infrastructure). The risk of postnatal MTCT (caused by the transmission of the virus through the breastfeeding) should be higher in countries with lower HDI (mostly using OPV) than in high-HDI countries (mostly using IPV) as WHO recommends continuation of breastfeeding in low- and middle-income countries, while it recommends formula feeding in high-income countries (23, 25). The observed protective effect of OPV in contrary to this existing higher risk of MTCT, in low- and middle-income countries was higher than that in countries with a relatively higher HDI. This means that the protective effect of OPV should be even higher than that we observed. The type of polio vaccine used in some countries may be switched from OPV to IPV. It might have some consequences. Depending on the HDI of the country, this switch may substantially affect the IncHIV1. For example, according to the model, in a country like Zambia with an HDI of 0.584, where use of OPV compared with IPV is associated with a 63% reduction in IncHIV1, switching the vaccine from OPV to IPV would be associated with an average 2.7-fold increase in IncHIV1 from 850 to more than 2000 infants per 100,000 children, despite an ART coverage of 75% in the country.

It might be thought that after implementation of several large international projects aimed at improving the ART coverage in areas where HIV/AIDS is prevalent (mostly low- and middle-income countries that commonly use OPV) (27, 28), the type of vaccine used in the model might be played as a surrogate for ART coverage in our model, and that it was the increased ART coverage in these endemic regions that protected the MTCT, rather than the use of OPV. ART coverage was not independently associated with IncHIV1 in our model. It might be though that this was because we also included the type of vaccine in the model; but, that was not the case. In fact, ART coverage was not significantly associated with IncHIV1 even if we had omitted the type of vaccine in our model (see Supplementary Materials).

OPV given to infants can cause significant shed in stool the attenuated poliovirus in the vaccine which is transmitted to their caregivers and other close contacts. This can initiate chains of transmission resulting in exposure of a significant number of people to the attenuated poliovirus, particularly in environments with poor sanitation (most probably countries with lower HDI) (29). This immunization through secondary exposure to the vaccine virus creates strong herd immunity against poliovirus, and may also contribute to higher resistance to other infections by stimulating the innate immunity (12, 15). The transmission rate of the virus throughout the community is expected to be lower in countries with higher sanitation and better health care infrastructures (most likely in high-income countries with higher HDI). This could be an additional reason why the protective effect of OPV was lower in countries with higher HDI.

If OPV exerts its protective effect through the stimulation of innate immune system, also called “trained immunity,” the protection provided is non-specific (5, 15). In a recent study, we have shown that use of OPV was associated with a 30% reduction in the incidence of COVID-19 (13). Another study conducted on almost 100,000 people in 1969–70, reported that OPV could decrease the incidence of influenza by 32% (10). Further studies should be done on the non-specific protection provided by OPV and other LAVs against other pathogens. This would be important for controlling other infectious diseases as well as the future pandemics.

## Limitations

One of the limitations of our study was that we did not consider the coverage of other LAVs in the studied countries. It has been shown that many of LAVs such as BCG and MMR, can provide non-specific protection against other infections (14, 15). However, the coverage of these vaccines was not much different in the studied countries. BCG is routinely administered to all people in almost 90% of countries in the world; in some countries only at-risk groups are vaccinated (30). Controlling these covariates is complicated, mainly because of the nature of our study. Ecological studies are mostly hypothesis-generating and no causal relationship could generally be inferred based on the results obtained from these studies.

## Conclusions

Use of OPV compared with IPV only for routine immunization was associated with a lower MTCT rate during the postnatal period. The protection provided was higher in low- and middle-income countries compared with high-income countries.

## Data Availability Statement

The original contributions presented in the study are included in the article/Supplementary Material, further inquiries can be directed to the corresponding author/s.

## Author Contributions

FH: conception of idea, data analysis, drafting the manuscript. FH, MY, and AS: data collection, interpretation of results, critical editing of the manuscript, final approval of the manuscript. All authors contributed to the article and approved the submitted version.

## Conflict of Interest

The authors declare that the research was conducted in the absence of any commercial or financial relationships that could be construed as a potential conflict of interest.

## Publisher's Note

All claims expressed in this article are solely those of the authors and do not necessarily represent those of their affiliated organizations, or those of the publisher, the editors and the reviewers. Any product that may be evaluated in this article, or claim that may be made by its manufacturer, is not guaranteed or endorsed by the publisher.

## Supplementary Material

Supplementary Table 1 Original Data.

Supplementary Data Sheet 1 R Program Codes.
