# Supplementary material for: Use of Oral Polio Vaccine and the Global Incidence of Mother-to-Child Human Immunodeficiency Virus Transmission
Source: Front Public Health. 2022 Jun 9;10:878298. doi: 10.3389/fpubh.2022.878298 (PMC9261940; doi:10.3389/fpubh.2022.878298)
Supplement: Supplementary Data Sheet 1 — R Program Codes. [file Data_Sheet_1.pdf]

SUPPLEMENTARY MATERIALS

Data Dictionary

IncHIV1	Incidence of HIV/AIDS in children aged <1 year (per 100,000 population)
PrevHIV	Prevalence of HIV/AIDS in women aged 15–49 years (per 100,000 population)
ARTCoV	Antiretroviral therapy coverage (% of people living with HIV)
HDI	Human development index
Vaccine	Type of polio vaccine used (OPV = 1, IPV = 0)

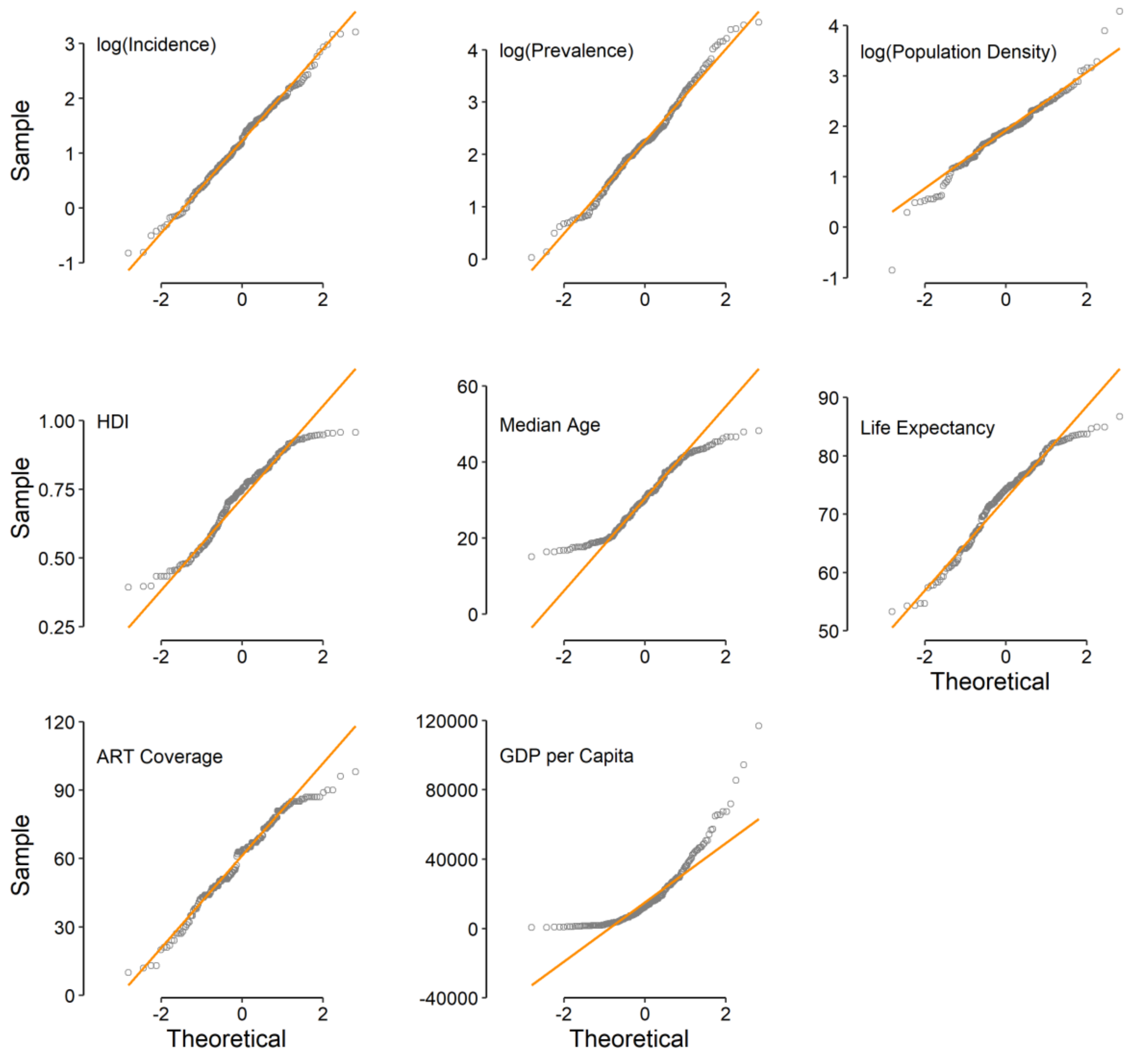

eFigure 1. QQ-plot of the eight studied continuous variables from the original dataset

Negative binomial regression model used in the current study using the original dataset:

```
fit <- glm.nb(IncHIV1 ~ PrevHIV + ARTCov + HDI * Vaccine, data = dat)
summary(fit)

Call:
glm.nb(formula = IncHIV1 ~ PrevHIV + ARTCov + HDI * Vaccine,
    data = dat, init.theta = 1.045632417, link = log)

Deviance Residuals:
    Min       1Q   Median       3Q      Max
-2.3303  -1.0175  -0.2929   0.1549   2.6054

Coefficients:
                 Estimate Std. Error z value Pr(>|z|)
(Intercept)     1.630e+01  3.470e+00   4.696 2.65e-06 ***
PrevHIV         1.867e-04  2.259e-05   8.264  < 2e-16 ***
ARTCov          4.223e-03  5.223e-03   0.809 0.418770
HDI            -1.679e+01  4.023e+00  -4.175 2.99e-05 ***
VaccineOPV     -1.138e+01  3.536e+00  -3.218 0.001293 **
HDI:VaccineOPV  1.463e+01  4.072e+00   3.593 0.000327 ***
---
Signif. codes:  0 '***' 0.001 '**' 0.01 '*' 0.05 '.' 0.1 ' ' 1

(Dispersion parameter for Negative Binomial(1.0456) family taken to be 1)

    Null deviance: 358.35  on 128  degrees of freedom
Residual deviance: 146.46  on 123  degrees of freedom
  (65 observations deleted due to missingness)
AIC: 1242.7

Number of Fisher Scoring iterations: 1


              Theta:  1.046
          Std. Err.:  0.124

 2 x log-likelihood:  -1228.729
```

Other models tested on the original dataset:

```
fit1 <- glm.nb(IncHIV1 ~ ARTCov, data = dat)
summary(fit1)

Call:
glm.nb(formula = IncHIV1 ~ ARTCov, data = dat, init.theta = 0.4717012032,
    link = log)

Deviance Residuals:
     Min        1Q    Median        3Q       Max
-2.30464  -1.24668  -0.63111  -0.06082   3.07873

Coefficients:
            Estimate Std. Error z value Pr(>|z|)
(Intercept) 3.504323   0.405270   8.647  < 2e-16 ***
ARTCov      0.017661   0.006522   2.708  0.00677 **
---
Signif. codes:  0 '***' 0.001 '**' 0.01 '*' 0.05 '.' 0.1 ' ' 1

(Dispersion parameter for Negative Binomial(0.4717) family taken to be 1)

    Null deviance: 173.86  on 130  degrees of freedom
Residual deviance: 166.00  on 129  degrees of freedom
  (63 observations deleted due to missingness)
AIC: 1397.1

Number of Fisher Scoring iterations: 1


              Theta:  0.4717
          Std. Err.:  0.0491

 2 x log-likelihood:  -1391.0820


#---
anova(fit, fit1)

Likelihood ratio tests of Negative Binomial Models

Response: IncHIV1
                             Model     theta Resid. df    2 x log-lik.   Test    df LR stat. Pr(Chi)
1                           ARTCov 0.4717012       129       -1391.082
2 PrevHIV + ARTCov + HDI * Vaccine 1.0456324       123       -1228.729 1 vs 2     6 162.3536       0


#---------------------
```

```
fit2 <- glm.nb(IncHIV1 ~ PrevHIV + ARTCov, data = dat)
summary(fit2)

Call:
glm.nb(formula = IncHIV1 ~ PrevHIV + ARTCov, data = dat, init.theta = 0.7938928518,
    link = log)

Deviance Residuals:
    Min       1Q   Median       3Q      Max
-2.4537  -1.1448  -0.3667   0.3415   2.4011

Coefficients:
              Estimate Std. Error z value Pr(>|z|)
(Intercept)  3.862e+00  3.148e-01  12.270   <2e-16 ***
PrevHIV      2.550e-04  2.331e-05  10.939   <2e-16 ***
ARTCov      -5.935e-03  5.178e-03  -1.146    0.252
---
Signif. codes:  0 '***' 0.001 '**' 0.01 '*' 0.05 '.' 0.1 ' ' 1

(Dispersion parameter for Negative Binomial(0.7939) family taken to be 1)

    Null deviance: 286.26  on 130  degrees of freedom
Residual deviance: 154.70  on 128  degrees of freedom
  (63 observations deleted due to missingness)
AIC: 1309.2

Number of Fisher Scoring iterations: 1


              Theta:  0.7939
          Std. Err.:  0.0896

 2 x log-likelihood:  -1301.2480


#---
anova(fit, fit2)

Likelihood ratio tests of Negative Binomial Models

Response: IncHIV1
                             Model     theta Resid. df    2 x log-lik.   Test    df LR stat.      Pr(Chi)
1                 PrevHIV + ARTCov 0.7938929       128       -1301.248
2 PrevHIV + ARTCov + HDI * Vaccine 1.0456324       123       -1228.729 1 vs 2     5 72.51905 3.064216e-14



#---------------------
fit3 <- glm.nb(IncHIV1 ~ PrevHIV + ARTCov + HDI, data = dat)
summary(fit3)

Call:
glm.nb(formula = IncHIV1 ~ PrevHIV + ARTCov + HDI, data = dat,
    init.theta = 0.9000587516, link = log)

Deviance Residuals:
    Min       1Q   Median       3Q      Max
-2.1650  -1.0335  -0.5131   0.1052   2.8155

Coefficients:
              Estimate Std. Error z value Pr(>|z|)
(Intercept)  6.344e+00  4.842e-01  13.102  < 2e-16 ***
PrevHIV      1.861e-04  2.425e-05   7.672 1.69e-14 ***
ARTCov      -1.940e-03  5.297e-03  -0.366    0.714
HDI         -3.943e+00  7.015e-01  -5.621 1.90e-08 ***
---
Signif. codes:  0 '***' 0.001 '**' 0.01 '*' 0.05 '.' 0.1 ' ' 1

(Dispersion parameter for Negative Binomial(0.9001) family taken to be 1)

    Null deviance: 310.83  on 128  degrees of freedom
Residual deviance: 150.02  on 125  degrees of freedom
  (65 observations deleted due to missingness)
AIC: 1262.5

Number of Fisher Scoring iterations: 1


              Theta:  0.900
          Std. Err.:  0.105

 2 x log-likelihood:  -1252.535


#---
anova(fit, fit3)

Likelihood ratio tests of Negative Binomial Models

Response: IncHIV1
                             Model     theta Resid. df    2 x log-lik.   Test    df LR stat.      Pr(Chi)
1           PrevHIV + ARTCov + HDI 0.9000588       125       -1252.535
2 PrevHIV + ARTCov + HDI * Vaccine 1.0456324       123       -1228.729 1 vs 2     2 23.80596 6.770201e-06
```

```
#---------------------
fit4 <- glm.nb(IncHIV1 ~ PrevHIV + ARTCov + HDI + Vaccine, data = dat)
summary(fit4)

Call:
glm.nb(formula = IncHIV1 ~ PrevHIV + ARTCov + HDI + Vaccine,
    data = dat, init.theta = 0.9660144946, link = log)

Deviance Residuals:
    Min       1Q   Median       3Q      Max
-2.2824  -0.9777  -0.3635   0.0903   3.6998

Coefficients:
              Estimate Std. Error z value Pr(>|z|)
(Intercept)  4.530e+00  7.717e-01   5.871 4.34e-09 ***
PrevHIV      1.851e-04  2.349e-05   7.878 3.33e-15 ***
ARTCov      -9.423e-04  5.321e-03  -0.177  0.85945
HDI         -2.719e+00  7.813e-01  -3.480  0.00050 ***
VaccineOPV   1.046e+00  3.196e-01   3.272  0.00107 **
---
Signif. codes:  0 '***' 0.001 '**' 0.01 '*' 0.05 '.' 0.1 ' ' 1

(Dispersion parameter for Negative Binomial(0.966) family taken to be 1)

    Null deviance: 332.43  on 128  degrees of freedom
Residual deviance: 148.60  on 124  degrees of freedom
  (65 observations deleted due to missingness)
AIC: 1253.5

Number of Fisher Scoring iterations: 1


              Theta:  0.966
          Std. Err.:  0.113

 2 x log-likelihood:  -1241.487


#---
anova(fit, fit4)

Likelihood ratio tests of Negative Binomial Models

Response: IncHIV1
                             Model     theta Resid. df    2 x log-lik.   Test    df LR stat.      Pr(Chi)
1 PrevHIV + ARTCov + HDI + Vaccine 0.9660145       124       -1241.487
2 PrevHIV + ARTCov + HDI * Vaccine 1.0456324       123       -1228.729 1 vs 2     1 12.75822 0.0003544487
```

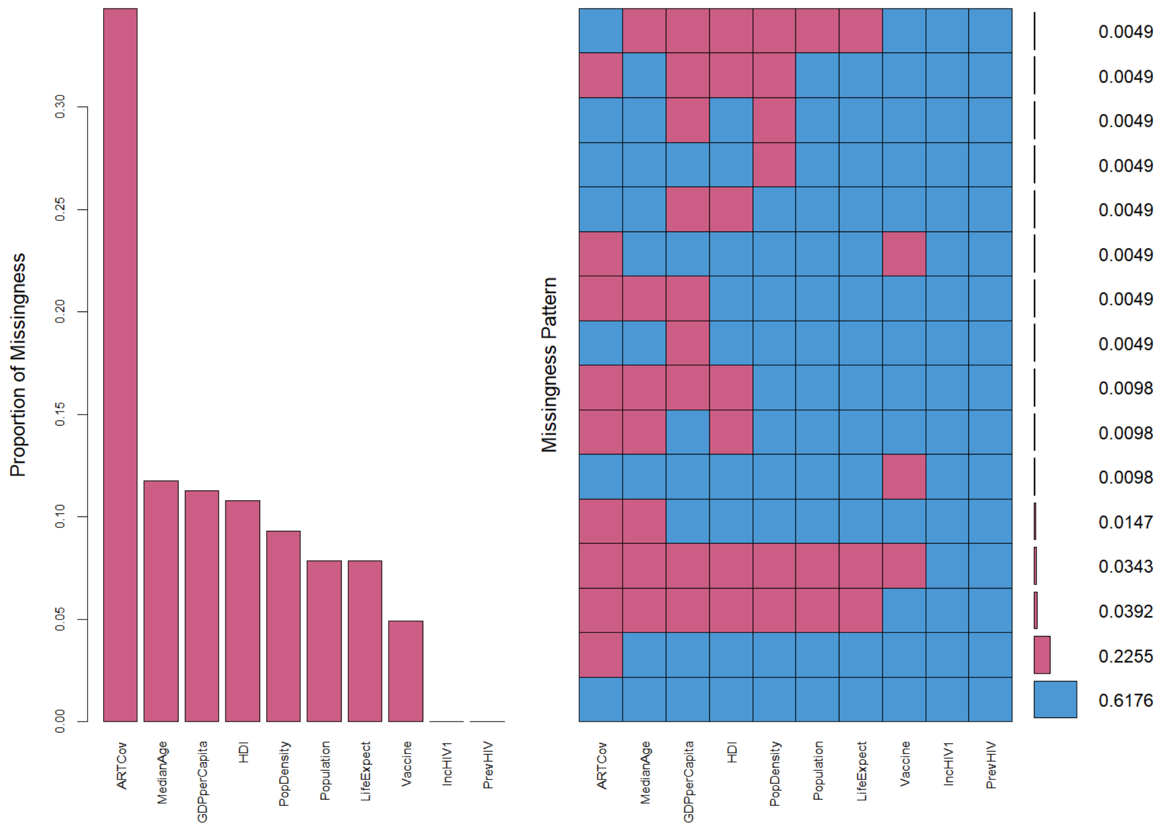

eFigure 2. Pattern of data missingness in the original dataset with 204 records. ARTCov is antiretroviral treatment coverage; MedianAge, median age; GDPperCapita, gross domestic product per capita; HDI, human development index; PopDensity, population density; LifeExpect, life expectancy; IncHIV1, incidence of HIV/AIDS in children aged <1 year; and PrevHIV, prevalence of HIV/AIDS in women aged 15–49 years. In the right panel, red indicates missingness. The numbers on the rightmost column are frequencies of each observed pattern.

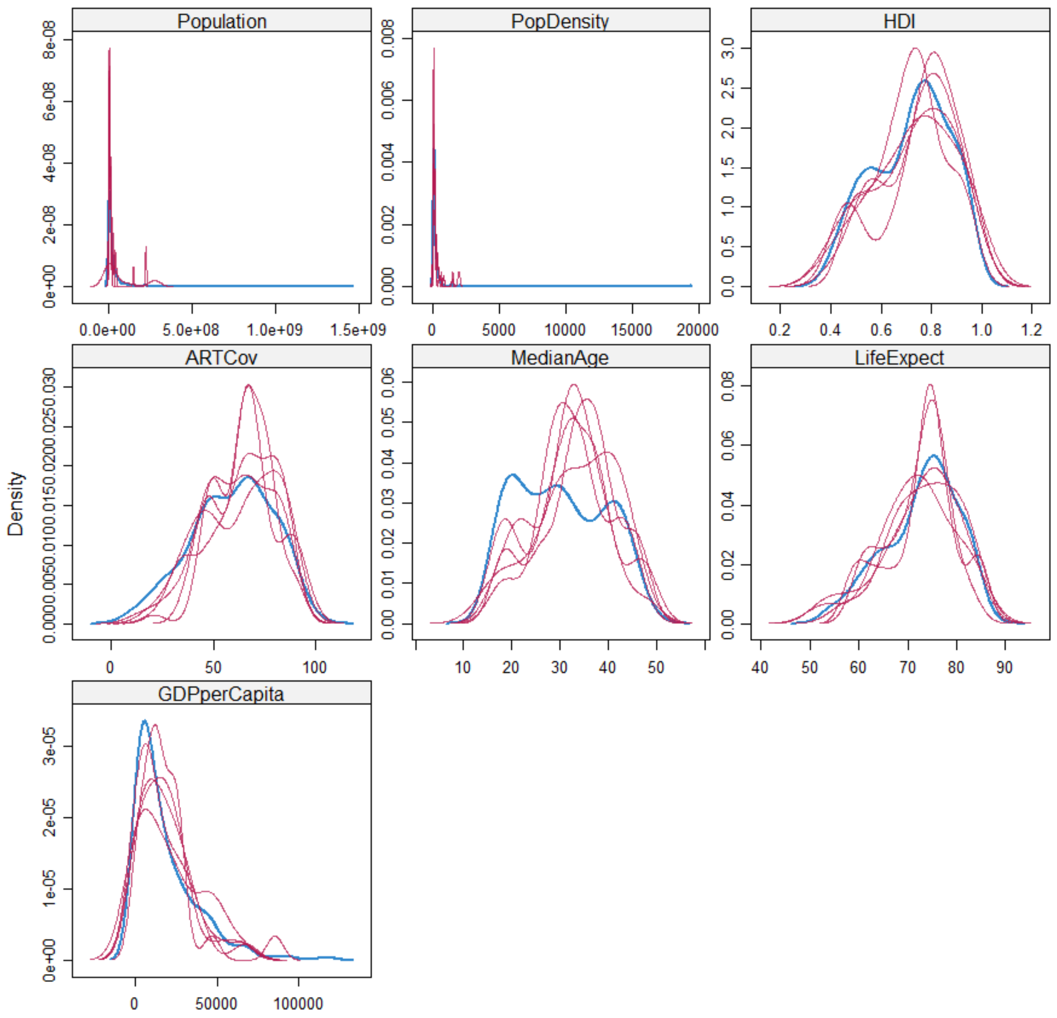

eFigure 3. The density of each of the five imputed datasets are shown in magenta; the density of the original observed dataset, in blue. PopDensity is population density; HDI, human development index; ARTCov, antiretroviral treatment coverage; MedianAge, median age; LifeExpect, life expectancy; and GDPperCapita, gross domestic product per capita.

Treating the missing values by multivariate imputation by chained equations:

```
dat <- read.csv("HIVdat.csv")    #-- The original dataset

#--- Setting imputation parameters
dat$Vaccine <- as.factor(dat$Vaccine)
init = mice(dat, maxit=0)
meth = init$method
predM <- quickpred(dat)

#--- Omitting Country, ISO, and the dependent variable, IncHIV1 for imputation
predM[, c("Country", "ISO", "IncHIV1", "PrevHIV")] = 0

imputed = mice(dat, method = meth, print = FALSE, predictorMatrix = predM, m = 5, seed = 2022)


#--- Pooled results of negative binomial regression model using the imputed datasets
imputedFit <- with(imputed, glm.nb(IncHIV1 ~ PrevHIV + ARTCov + HDI * Vaccine))
summary(pool(imputedFit))

#--- Pooled results
            term      estimate    std.error  statistic       df      p.value
1    (Intercept) 10.1265287341 1.979916e+00  5.1146266 64.37624 3.046191e-06
2        PrevHIV  0.0001738702 1.947907e-05  8.9260034 91.44547 4.352074e-14
3         ARTCov  0.0026706504 5.293817e-03  0.5044848 48.77487 6.161923e-01
4            HDI -9.3195932819 2.319146e+00 -4.0185450 57.26902 1.728969e-04
5     VaccineOPV -5.0120788801 2.060728e+00 -2.4321887 58.55524 1.808477e-02
6 HDI:VaccineOPV  6.8973208132 2.422286e+00  2.8474433 55.33256 6.173531e-03
```
